# Rapid loss of flight in the Aldabra white-throated rail

**DOI:** 10.1371/journal.pone.0226064

**Published:** 2019-12-23

**Authors:** Janske van de Crommenacker, Nancy Bunbury, Hazel A. Jackson, Lisa J. Nupen, Ross Wanless, Frauke Fleischer-Dogley, Jim J. Groombridge, Ben H. Warren

**Affiliations:** 1 Seychelles Islands Foundation (SIF), Mont Fleuri, Victoria, Mahé, Seychelles; 2 Durrell Institute of Conservation and Ecology (DICE), School of Anthropology and Conservation, University of Kent, Canterbury, Kent, United Kingdom; 3 Centre for Ecology and Conservation, University of Exeter, Penryn, United Kingdom; 4 DST/NRF Centre of Excellence at the Percy FitzPatrick Institute of African Ornithology, University of Cape Town, Cape Town, South Africa; 5 Institute of Marine Affairs and Resources Management, National Taiwan Ocean University, Keelung, Taiwan; 6 Institut de Systématique, Evolution, Biodiversité (ISYEB), Muséum National d'Histoire Naturelle, Sorbonne Universités, Paris, France; Temple University, UNITED STATES

## Abstract

Flight loss has evolved independently in numerous island bird lineages worldwide, and particularly in rails (Rallidae). The Aldabra white-throated rail (*Dryolimnas* [*cuvieri*] *aldabranus*) is the last surviving flightless bird in the western Indian Ocean, and the only living flightless subspecies within *Dryolimnas cuvieri*, which is otherwise volant across its extant range. Such a difference in flight capacity among populations of a single species is unusual, and could be due to rapid evolution of flight loss, or greater evolutionary divergence than can readily be detected by traditional taxonomic approaches. Here we used genetic and morphological analyses to investigate evolutionary trajectories of living and extinct *Dryolimnas cuvieri* subspecies. Our data places *D*. [*c*.] *aldabranus* among the most rapid documented avian flight loss cases (within an estimated maximum of 80,000–130,000 years). However, the unusual intraspecific variability in flight capacity within *D*. *cuvieri* is best explained by levels of genetic divergence, which exceed those documented between other volant taxa versus flightless close relatives, all of which have full species status. Our results also support consideration of *Dryolimnas* [*cuvieri*] *aldabranus* as sufficiently evolutionary distinct from *D*. *c*. *cuvieri* to warrant management as an evolutionary significant unit. Trait variability among closely related lineages should be considered when assessing conservation status, particularly for traits known to influence vulnerability to extinction (e.g. flightlessness).

## Introduction

Organisms living in island environments frequently undergo remarkable evolutionary changes [1–4]. One such change is loss of flight, which has occurred worldwide in 26 bird families from 17 orders [5]. Flight enables organisms to disperse, escape from predators and forage [e.g., 6,7]. Species-poor islands that naturally lack mammal and bird predators have been important in the evolution of flightlessness [8,9]. Consequently, loss of flight has evolved independently in many insular bird species worldwide. Despite the high incidence of avian flight loss on islands [8], the pace of evolutionary transitions underlying this trait is poorly known. This is at least partly due to the fact that many insular flightless or poorly volant bird species are extinct, and the scarcity of cases [5,10–12] in which there exist gradations in flightlessness among or within extant lineages.

The avian family with the highest incidence of flight loss worldwide is the Rallidae (rails; Order Gruiformes), with over 25% of the extant rail species being flightless [13]. The family includes an estimated 135–150 extant species, plus numerous extinct forms [14], with a global distribution that includes many oceanic islands, and a high proportion of island endemics [15].

Flightlessness has contributed to high extinction rates of island birds in the last 50,000 years, primarily driven by human colonization and the concomitant introduction of non-native predators [16]. Rallidae have probably been the most susceptible avian family in this regard. At least 65 species of Rallidae worldwide are documented as late Quaternary extinctions [17,18] and another 35 species as recent extinctions (since *ca*. 1500 years BP). However, it is estimated that such documented cases are greatly outnumbered by undocumented human-induced rail extinctions, which may total 2000 species in the Pacific islands alone [16,19]. Appropriate conservation assessment and protection of the remaining flightless Rallidae and other avian species is therefore vital.

**Fig 1:**
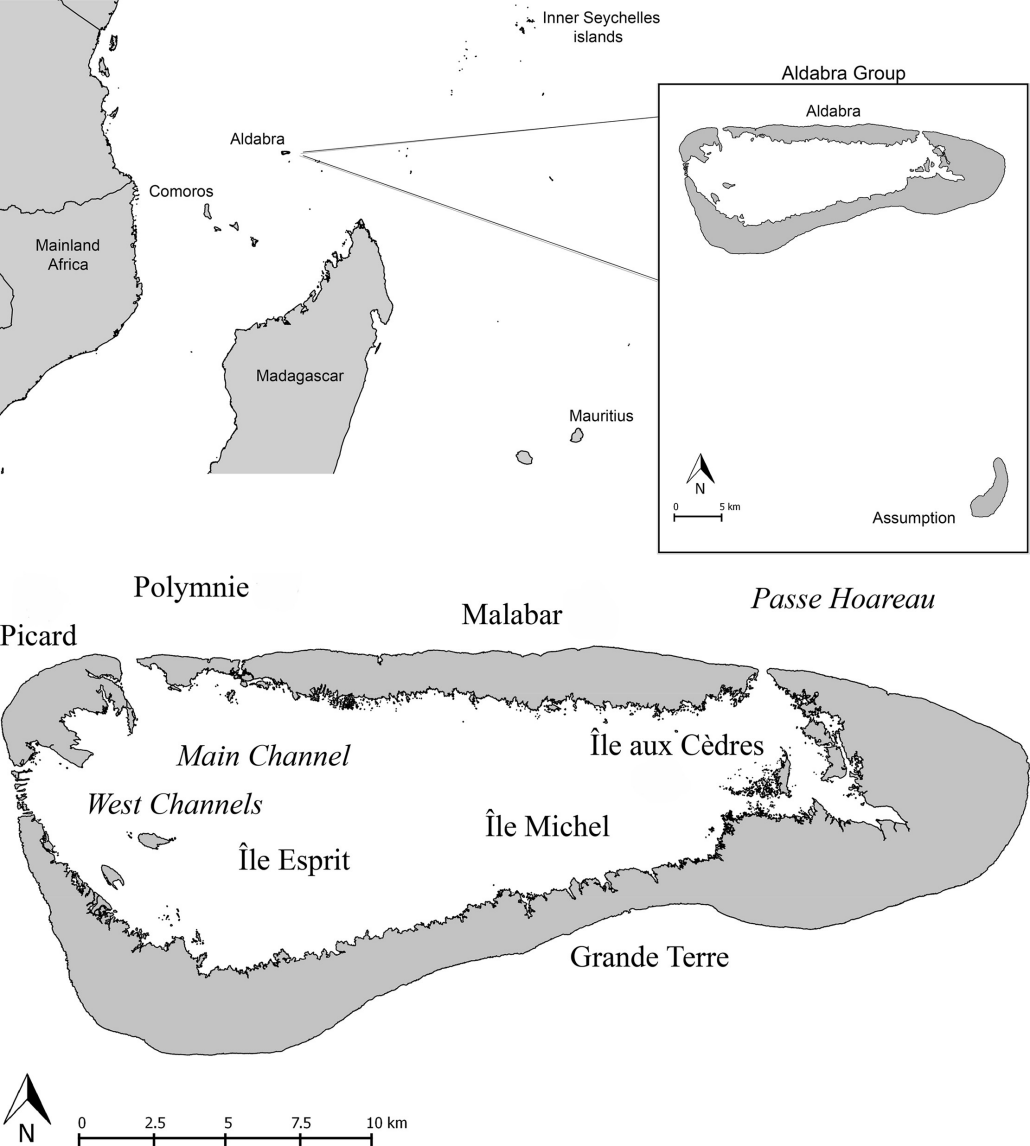
(A) Western Indian Ocean with Madagascar, Aldabra Atoll and Assumption Island (the latter two enlarged in the inset), and (B) the islands of Aldabra Atoll, of which Picard, Malabar and Polymnie are populated by *D*. [*c*.] *aldabranus*, as was Île aux Cèdres until recently.

Our research focuses on the last surviving flightless bird in the biodiversity hotspot of the Western Indian Ocean [20,21]: the Aldabra white-throated rail (*Dryolimnas* [*cuvieri*] *aldabranus*), which occurs only on Aldabra Atoll in the southern Seychelles. Historically, *D*. *cuvieri* occurred on all four islands of the Aldabra group–Aldabra, Assumption (Fig 1), Cosmoledo and Astove–before being extirpated from the latter three [12, 22–24]. There are two other recognised subspecies: the volant Madagascar white-throated rail *D*. *c*. *cuvieri*, a common endemic to Madagascar [12,22], and the extinct Assumption rail (*D*. *c*. *abbotti*), endemic to Assumption [25,26]. A second, extinct species of *Dryolimnas*, *D*. *augusti*, was recently described based on fossil remains from Réunion Island [27], and a third species, flightless and now extinct, once occurred on Mauritius [28,29]. Based on existing knowledge and applying the common assumption that taxonomic status reflects genetic divergence, the flightless Aldabra rail subspecies represents an enigma–it is flightless, yet only considered a subspecies in an otherwise volant species. Therefore, either it would appear to be a candidate for the youngest documented fully flightless bird lineage worldwide (and potential example of such an evolutionary change being very rapid; [28]), or it is more divergent from the Madagascar lineage than is readily inferred from current taxonomy.

Here we use genetic data from modern samples and museum specimens to examine the phylogenetic placement of the flightless lineages of the Aldabra group, and investigate whether or not their closest relative is indeed *Dryolimnas* of Madagascar. We further use these data, in combination with morphological data from modern and museum samples, to assess the degree of divergence of the flightless *D*. [*c*.] *aldabranus* and the poorly volant *D*. *c*. *abbotti* from the volant lineage of Madagascar. Genetic variation among populations of *D*. [*c*.] *aldabranus* is used to refine our understanding of important dispersal events in the biogeographic history of this lineage. We also show how differentiation among *D*. [*c*.] *aldabranus* subpopulations can be used to inform effective management of this unique bird, the last survivor among 12–17 flightless avian lineages that once occupied the Western Indian Ocean region before human arrival [24].

## Materials and methods

### Ethics statement

The ethical guidelines promoted by the Association for the Study of Animal Behaviour were followed. Permission for sampling on Aldabra was issued by the Seychelles Islands Foundation (local management authority), and the Department of Environment and the Seychelles Bureau of Standards approved all research activities (approval reference A0347). Sequences have been submitted to the NCBI GenBank (Accession Numbers: MH614934–MH614960, MH645373–MH645415 and MH651394–MH651440).

### Study site and species

The total population of *D*. [*c*.] *aldabranus* occurs in an area of *ca*. 37.2 km^2^, on the raised atoll of Aldabra (152.6 km^2^, 9°24' S, 46°20' E; Fig 1): with subpopulations on Picard (area: 9.4 km^2^), Polymnie (1.9 km^2^) and Malabar (25.9 km^2^). A UNESCO World Heritage Site since 1982, Aldabra has been managed entirely for research and conservation since 1979 with only a very small resident human population.

The Picard subpopulation of *D*. [*c*.] *aldabranus* originates from a successful reintroduction of 18 rails from Malabar in 1999 [30], after introduced feral cats on Picard were removed by humans in the 1970s [30]. For the sake of clarity regarding origin, we refer hereafter to these recently translocated rails on Picard with the term 'Malabar*'. This subpopulation has since expanded to more than 2500 individuals [31]. *Dryolimnas* [*c*.] *aldabranus* also occurred until very recently on the smaller islet of Île aux Cèdres, and was reportedly more morphologically distinct (leg and bill size) from *D*. [*c*.] *aldabranus* on other islands than was *D*. *c*. *abbotti* [23,25]. A recent extensive survey (Seychelles Islands Foundation (SIF), unpubl. data) indicated that this subpopulation is probably extinct (last confirmed sighting in 2000; Wanless, pers. obs.). The original *D*. [*c*.] *aldabranus* subpopulations on Grande Terre and Picard were extirpated (*ca*. mid-1800s and *ca*. 1910, respectively) following the introduction of feral cats [22,32, but see 25].

*Dryolimnas c*. *abbotti* was historically common on Assumption (~11 km^2^; Fig 1), and was also well on its way to becoming flightless (i.e. being poorly volant, [22]), but had become extinct by 1937 [23,33,34], presumably due to the introduction of mammalian predators [11,30].

The volant *D*. *c*. *cuvieri* of Madagascar shows a stable population trend over its *ca*. 854,000 km^2^ range [35], and is considered common [36], although no reliable population estimates are available. It occupies various habitats throughout Madagascar, including forest, wetlands, mangroves, beaches and rice paddy-fields [37].

### Sample collection

Thirty-eight samples (S1 Appendix), representing all three *Dryolimnas* subspecies were analysed (including 19 historical toe pad samples from museum specimens, and 19 contemporary blood samples from living birds): 25 *D*. [*c*.] *aldabranus* samples (six historical, 19 contemporary), four *D*. *c*. *abbotti* (all historical), and nine *D*. *c*. *cuvieri* from different locations in Madagascar (all historical). The samples include individuals from all *D*. [*c*.] *aldabranus* subpopulations, except the extinct Grande Terre subpopulation, for which no museum specimens exist. Specimens from the extinct Picard subpopulation were available from museum skins. The 19 contemporary *D*. [*c*.] *aldabranus* blood samples were collected on Aldabra (Polymnie: *n* = 7, Malabar: *n* = 5, Malabar*: *n* = 4 and Île aux Cèdres: *n* = 3) in two periods (years 2000 [Île aux Cèdres] and 2011–2014). We used only historical *D*. *c*. *cuvieri* samples after attempts to obtain contemporary samples were unsuccessful (i.e., despite several requests to different local researchers, nobody could provide us with samples).

### DNA isolation, amplification and sequencing

DNA was extracted (S2 Appendix) using a Bioline Isolate Genomic DNA extraction kit (Bioline, UK), following the manufacturer's standard protocols for blood (contemporary samples) and tissue (museum samples). The museum samples had a range of ages dating back to the 1870s (S1 Appendix), and potentially low endogenous DNA concentration. They were therefore treated in a dedicated museum DNA laboratory. From each sample, 593bp from the mitochondrial regions Control Region (CR; 306bp) and Cytochrome b (Cytb; 287bp) was amplified and sequenced (Table 1, S2 Appendix). Negative controls were included to check the absence of contamination during the extraction and PCR process. For historical samples, amplifications were conducted using a suite of short overlapping fragment primers designed for this study with the NCBI Primer designing tool (http://www.ncbi.nlm.nih.gov/tools/primer-blast/; Table 1). PCR products were sequenced by Macrogen-South Korea and Macrogen-Europe. Sequence reads were manually checked and then aligned and edited using the programme FinchTV 1.4 (Geospiza), BioEdit 7.2.0 [38] and CodonCode Aligner 4.2.4 (CodonCode Corporation, Dedham, MA). Consensus sequences were aligned using the programme ClustalX 2.1.12 [39], and the genes were concatenated using Sequencematrix [40].

**Table 1:** Primers and experimental conditions used to amplify and sequence the genes (in contemporary and historical samples) used in this study.

					PCR conditions			
Contemporary specimens								
DNA type	Gene region	Primer names	Sequence 5' - 3'	Source	Nr of cycles	Denaturation	Annealing	Extension
Mitochondrial DNA	Cytochrome b	L14841	AAAAGCTTCCATCCAACATCTCAGCATGATGAAA	[41]	40	95°C for 15 sec	58°C for 15 sec	72°C for 10 sec
		H15156	AAACTGCAGCCCCTCAGAATGATATTT					
	Control Region	RailCRcompSPEC-f	GCGTACCCCCTACTTTCAAGG	Own design	33	95°C for 15 sec	56°C for 15 sec	72°C for 10 sec
		RailCRcompSPEC-r	GACCGAGGAACCAGAGGC					
**Historical specimens**								
	Gene region	Primer names	Sequence 5' - 3'	Source	Nr of cycles	Denaturation	Annealing	Extension
Mitochondrial DNA	Cytochrome b	Cytb/1 (f & r) (96 bp)	GCACTACACTGCAGACACAA (f) & TTAGCGTGGAGGTTGCGG (r)	Own design	35	95°C for 15 sec	55°C for 15 sec	72°C for 10 sec
		Cytb/2 (f & r) (114 bp)	CACATGCCGCAACGTACAAT (f) & GAGCCGTAGTAGAATCCTCGG (r)					
		Cytb/3 (f & r) (132 bp)	GCCGAGGATTCTACTACGGCTC (f) & CCCCTCAGAATGATATTTGTCCTCA (r)					
	Control Region	For *D*. [*c*.] *aldabranus* and *abbotti*: RailCRcompSPEC (f & r) (351 bp)	See modern DNA primer	Own design	35	95°C for 15 sec	58°C for 15 sec	72°C for 10 sec
		For *D*. *c*. *cuvieri*: MadRailCR (f & r) (351 bp)	See modern DNA primer	Own design	35	95°C for 15 sec	58°C for 15 sec	72°C for 10 sec

* All PCR amplifications were started with an initial denaturation step of 1 min at 95°C before commencing the cycles.

### Data partition, model selection and phylogenetic inference

For the concatenated mitochondrial dataset (593bp), the program Partitionfinder [42] was used to test the congruence of phylogenetic signal from the different genes and determine the optimal substitution models of nucleotide evolution for each partition, according to Bayesian information criteria (BIC). The HKY + gamma evolutionary model was found to be the optimal model, and was used for the estimation of the time-calibrated phylogeny.

### Molecular-based estimates of divergence: Time calibrated phylogenetic reconstruction

Time-calibrated phylogenies were reconstructed using Beast v.1.8.2 [43] via the CIPRES Science Gateway [44]. Sequences from GenBank of Rallidae closely related to *Dryolimnas–Lewinia pectoralis*, *L*. *mirifica*, *L*. *muelleri*, *Gallirallus philippensis*, and two subspecies of *G*. *australis*–were selected as outgroups based on the phylogeny of Garcia-R *et al*. [45].

The following calibrations were specified: time to most common recent ancestor (TMRCA) of 2.588 Myr for the divergence of *Dryolimnas* and *Lewinia*, and 0.125 Myr for the most recent emergence of the Aldabra group. Our reasoning behind this choice of calibration dates was as follows:

1) The densely sampled phylogeny of Rallidae in Garcia–R. *et al*. [45] demonstrates that *Crex crex* shares a clade with *Lewinia* and *Dryolimnas* that gains 97% bootstrap support. Our phylogeny is fully congruent with that in Garcia–R. *et al*. [45]. A fossil *Crex crex* demonstrates that this taxon is at least 2.588 million years (Myr) old (http://fossilworks.org/bridge.pl). By deduction, the divergence of *Lewinia* and *Dryolimnas* in our tree must also be at least 2.588 Myr, and we calibrated it accordingly. 2) The estimated last emergence of the Aldabra group 0.125 ± 0.02 Ma ago [46] provided an upper bound estimate for the divergence of the common ancestor of *D*. *c*. *cuvieri* and the Aldabra group taxa (*D*. [*c*.] *aldabranus* and *abbotti*).

Some of the nodes we seek to date involve inter-specific relationships, while others may be intra-specific. Therefore, we compared results under the Yule speciation tree prior [47] with coalescent tree priors. Furthermore, we know that *D*. *cuvieri* has declined in population size (most severely on Aldabra) in historical times, but have no data on the nature of this decline. Therefore, under a coalescent tree prior we compared outputs with an inversegamma prior on population size dynamics, versus a uniform prior, assuming a constant unknown population size through time.

For each of the three alternative tree priors (Yule, Coalescent-Uniform, and Coalescent-Inversegamma), a lognormal relaxed clock was used with lognormal distributions for the calibration priors, and two replicate Monte Carlo Markov chains (MCMC) were performed for 10 million generations, sampling every 1000 generations under an HKY + gamma evolutionary model [48]. Mixing was confirmed by examining effective sample sizes (ESS>200) for all parameters using Tracer v1.6.0 [49]. Trees from the first 10% of generations were discarded as burn-in and a maximum clade credibility tree was summarised in TreeAnnotator v1.8.2 [43] and visualised in FigTree v1.4.2 [50]. After checking the convergence of Bayesian analyses through the congruence of outputs from replicate chains (under each alternative tree prior), two final MCMCs (Yule & Coalescent-Inversegamma tree priors) were performed for 30 million generations following the same protocol as for earlier chains.

### Phylogenetic relationships: Hypothesis testing

In addition to our Bayesian analyses, a best-scoring Maximum-Likelihood tree was reconstructed using RAxML v. 8.2.8 [51] under the GTR + G substitution model. Clade support was measured with the rapid bootstrap algorithm [52] using 5000 replicates. Furthermore, using the Shimodaira and Hasegawa (SH) test [53] implemented in PAUP*, we checked the monophyly of rail populations and discriminated between alternative scenarios of island colonization. Using the concatenated dataset, the SH test was used to compare the optimal Bayesian topology with topologies constrained to correspond to alternative hypotheses reconstructed using parsimony (heuristic searches, holding one tree at each step).

### Haplotype networks

Median-joining haplotype networks were constructed (POPART v1.7; [54]) both for the concatenated mtDNA dataset, and for each marker separately, using the setting epsilon = 0 (minimum spanning network).

### Morphological analyses

Morphological measurements (wing and tail length [using a flat ruler], tarsus length, bill length [bill tip to nasofrontal hinge], bill width and height [both measured at centre of nostrils]) were taken from all live birds and museum specimens. However, museum specimens tend to shrink upon drying [55] which compromises their reliability for comparison with live birds [56]. Therefore, only measurements from museum specimens were used for our morphological analyses. Measurements from museum specimens that were not genetically sampled were included to increase the sample size. To identify morphological differentiation between subspecies, a discriminant function analysis was performed in SPSS v25 (IBM). All traits were analysed separately with general linear models, with subspecies and sex as factors in the model. As a test for the homogeneity of slopes, the interaction between subspecies and sex were tested. Stepwise elimination was performed when the interaction and sex were found to be non-significant.

## Results

### Phylogenetic relationships, divergence times and genetic distances

**Fig 2:**
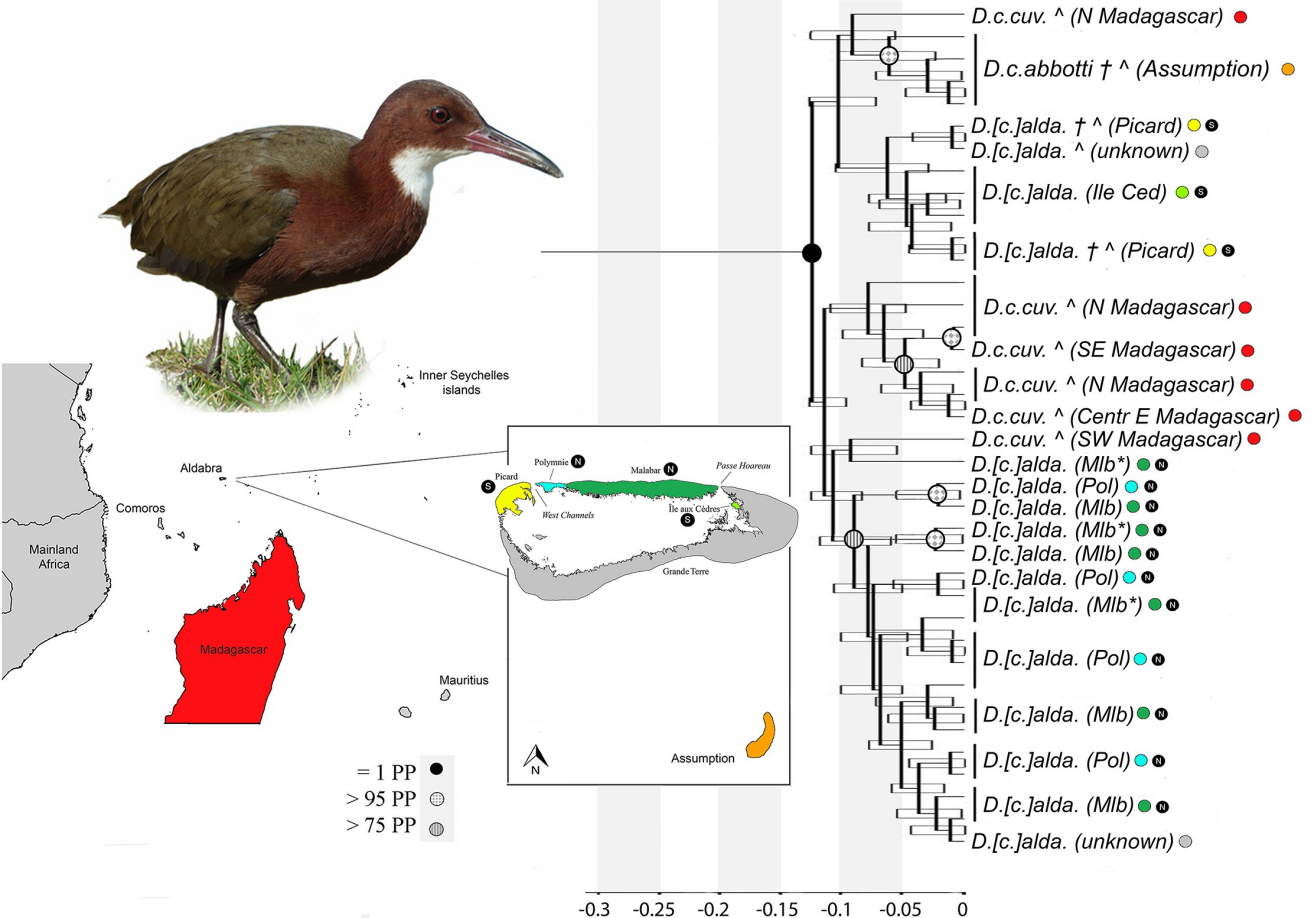
Bayesian analysis (Yule speciation prior, 30 million generations) of concatenated Cytb and CR mtDNA data from contemporary and museum (indicated with ^) specimens of *D*. *c*. *cuvieri* from Madagascar, *D*. *c*. *abbotti* from Assumption, and *D*. [*c*.] *aldabranus* from Aldabra (different islands; indicated with colours, and Native North (N) and South (S) Aldabra islands are indicated with the black encircled letters). Bayesian branch support values (>75%) are indicated. Error bars display the 95% higher posterior density and time on the x-axis is given in millions of years before the present. († = population now extinct, Mlb* = Picard population recently introduced from Malabar). Although the analysis with the Yule speciation prior was illustrated here because of the interspecific nature of our deeper-level sampling (see [57] for discussion), the equivalent analyses with Coalescent-Inversegamma and Coalescent-Uniform speciation priors are illustrated in S3 Appendix. Furthermore, to magnify nodes and confidence intervals of interest for our focus, we excluded the outgroups from this figure. The full tree (Yule speciation prior) including the outgroups can also be found in S3 Appendix.

Tree topology is highly concordant between Bayesian and ML analyses, between Bayesian analyses with different tree priors, and among replicate Bayesian analyses with the same tree prior. Bayesian analyses converged, with date estimates for supported (PP≥0.95) ingroup nodes varying by a maximum of 2.6% (1600 years) between replicate chains. Based on Bayesian analyses of 10 million generations, divergence time estimates show consistent variation depending on the tree prior used (Yule estimates being older than Coalescent-Uniform estimates, and Coalescent-Uniform estimates being older than Coalescent-Inversegamma estimates). We therefore selected Yule and Coalescent-Inversegamma tree priors for our final two Bayesian analyses (each was run for 30 million generations), thereby obtaining longer chains for the two tree priors that cover the full range of divergence estimates based on findings from shorter chains. Our Bayesian analyses (Fig 2) confirm that the flightless and poorly volant taxa of the Aldabra group (*D*. [*c*.] *aldabranus* and *D*. *c*. *abbotti* subspecies, respectively) are indeed most closely related to the volant white-throated rail of Madagascar (*D*. *c*. *cuvieri*; PP = 1.0 for the monophyly of *D*. *cuvieri*). Although phylogenetic relationships are congruent with existing taxonomy in this respect, levels of genetic divergence are somewhat incongruous, with substantial genetic divergence within *Dryolimnas cuvieri;* the highest absolute sequence divergence of 2.1% between flightless *D*. [*c*.] *aldabranus* and volant *D*. *c*. *cuvieri* in Cytb, and 1.7% divergence between *D*. [*c*.] *aldabranus* and poorly volant *D*. *c*. *abbotti*, exceeds the minimum absolute divergence of 1.2% in Cytb encountered between other flightless and volant rail taxa, all of which have full species status [41]. Mean nucleotide divergences between the *Dryolimnas* subspecies derived from the concatenated (593 bp) mtDNA dataset are noteworthy in three cases (Table 2) between: 1) *D*. [*c*.] *aldabranus* and *D*. *c*. *cuvieri*; 2) *D*. *c*. *abbotti* and *D*. [*c*.] *aldabranus*; and 3) *D*. [*c*.] *aldabranus* populations native to the southern islands of Aldabra (Île aux Cèdres and Picard; putatively-extinct and extinct populations; herein “Native South Aldabra”) and *D*. [*c*.] *aldabranus* populations native to northern islands of Aldabra (Malabar, Malabar* and Polymnie; extant; herein “Native North Aldabra”).

**Table 2:** Divergences of the different populations/(sub)species of *D*. *cuvieri* for Cytb and CR combined, and for Cytb alone. The genetic distance metric used is absolute distance.

Comparison of *D*. *cuvieri*	Gene	Pairwise substitutions	Genetic distance
*D*. [*c*.] *aldabranus* vs *D*. *c*. *cuvieri*	Cytb-CR	3–9	0.51–1.5%
	Cytb alone	0–6	0–2.1%
*D*. *c*. *abbotti* vs *D*. *c*. *cuvieri*	Cytb-CR	4–8	0.67–1.3%
	Cytb alone	0–1	0–0.35%
*D*. [*c*.] *aldabranus* vs *D*. *c*. *abbotti*	Cytb-CR	2–9	0.34–1.5%
	Cytb alone	0–5	0–1.7%
*D*. [*c*.] *aldabranus*: Île aux Cèdres from Native North Aldabra (Malabar-Malabar*-Polymnie)	Cytb-CR	3–9	0.51–1.5%
	Cytb alone	0–7	0–2.4%
*D*. [*c*.] *aldabranus*: Picard (extinct) from Native North Aldabra	Cytb-CR	3–6	0.51–1%
	Cytb alone	0–5	0–1.7%

Malabar* = Picard population recently introduced from Malabar

Our relaxed clock analysis suggests that the divergence of Aldabra and Assumption populations from those on Madagascar occurred *ca*. 0.07–0.13 Myr ago. The Assumption population (*D*. *c*. *abbotti*) forms a monophyletic group within the species *Dryolimnas cuvieri* (>95% posterior probability under both Yule & Coalescent-Inversegamma tree priors). Tree topology is consistent with a lack of monophyly for all other subspecies; e.g., *D*. [*c*.] *aldabranus* populations from Malabar, Malabar* and Polymnie (“Native North Aldabra”; Fig 1) do not form a monophyletic group with *D*. [*c*.] *aldabranus* on Île aux Cèdres and Picard (“Native South Aldabra”; Fig 1) in any of the Bayesian analyses, nor in our ML analysis. However, all the relevant nodes lack significant branch support (i.e., ≥70% bootstrap values, ≥95% posterior probability regardless of tree prior). The SH test did not allow us to reject hypotheses of monophyly for each of the three major *D*. *cuvieri* populations: Aldabra group (i.e., Aldabra and Assumption), p = 0.19; Aldabra, p = 0.17; and Madagascar, p = 0.18). Therefore, signal in our CR and Cytb data neither provides significant support for nor against the monophyly of these populations–both scenarios remain plausible.

### Haplotype networks

**Fig 3:**
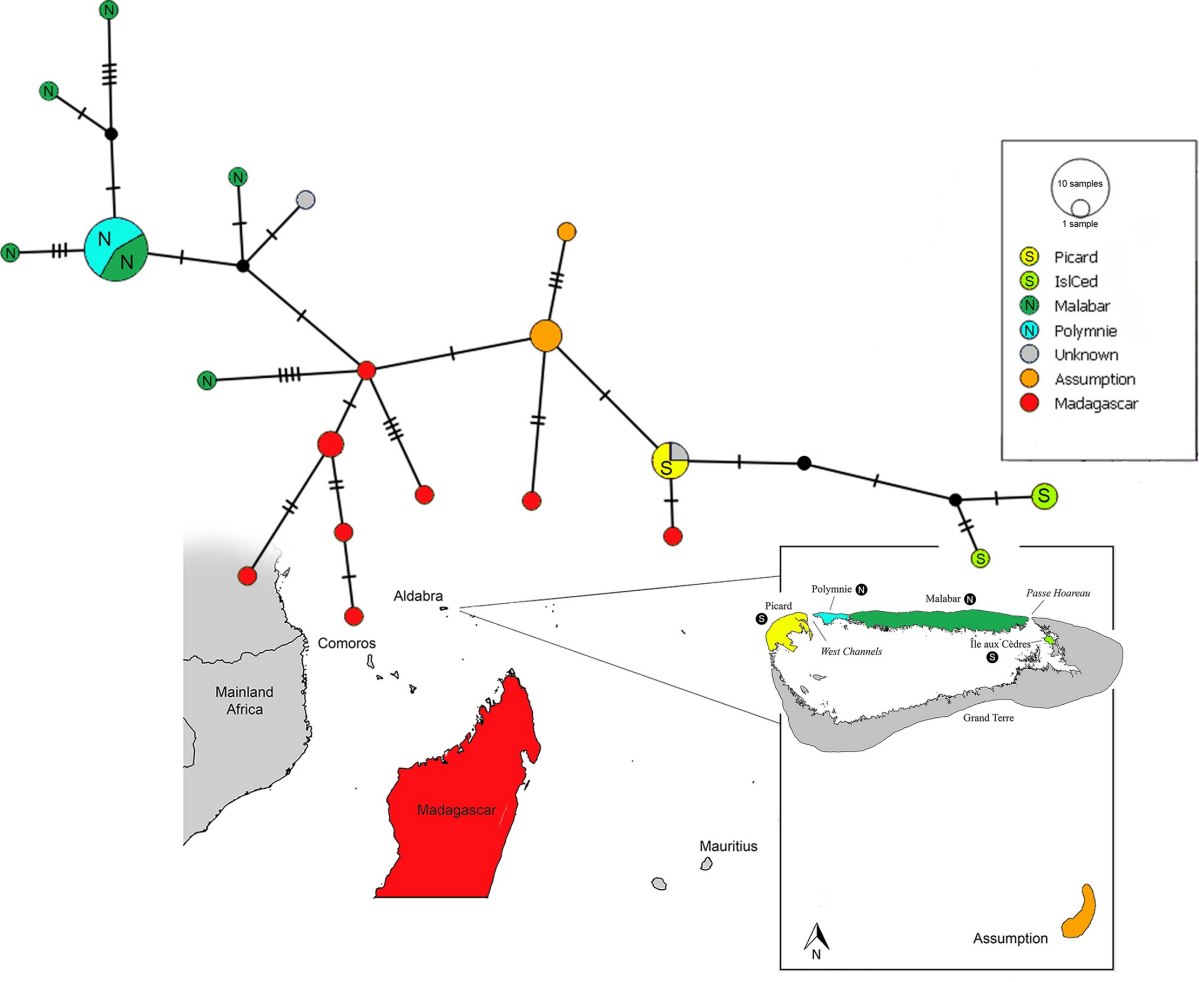
Median-joining haplotype networks for concatenated mtDNA (including CR and Cytb). For the Aldabra rail, the individuals from Malabar and Malabar* are pooled. Native North (N) and South (S) Aldabran islands are indicated with the encircled letters. Median-joining haplotype networks for each of the separate markers can be found in S4 Appendix.

Haplotype networks (Fig 3, S4 Appendix) show substantial genetic variation of *D*. *cuvieri* within the Aldabra group. *Dryolimnas c*. *cuvieri* of Madagascar is intermediate between two groups of *D*. [*c*.] *aldabranus* on each side of the network. This pattern in the concatenated mtDNA network (Fig 3) reflects divergence in the CR, rather than in Cytb (S4 Appendix). Distinct from *D*. *c*. *cuvieri* specimens is a major haplotype grouping represented mostly by contemporary specimens of *D*. [*c*.] *aldabranus* from Native North Aldabra (Fig 3). Another major grouping consists of historical *D*. [*c*.] *aldabranus* specimens from Native South Aldabra, with *D*. *c*. *abbotti* between these haplotypes and those of Madagascar (Fig 3). The haplotype networks also indicate that *D*. *c*. *abbotti* has undergone fewer mutational changes relative to the Madagascar population than any of the *D*. [*c*.] *aldabranus* subpopulations.

### Morphological analyses

**Fig 4:**
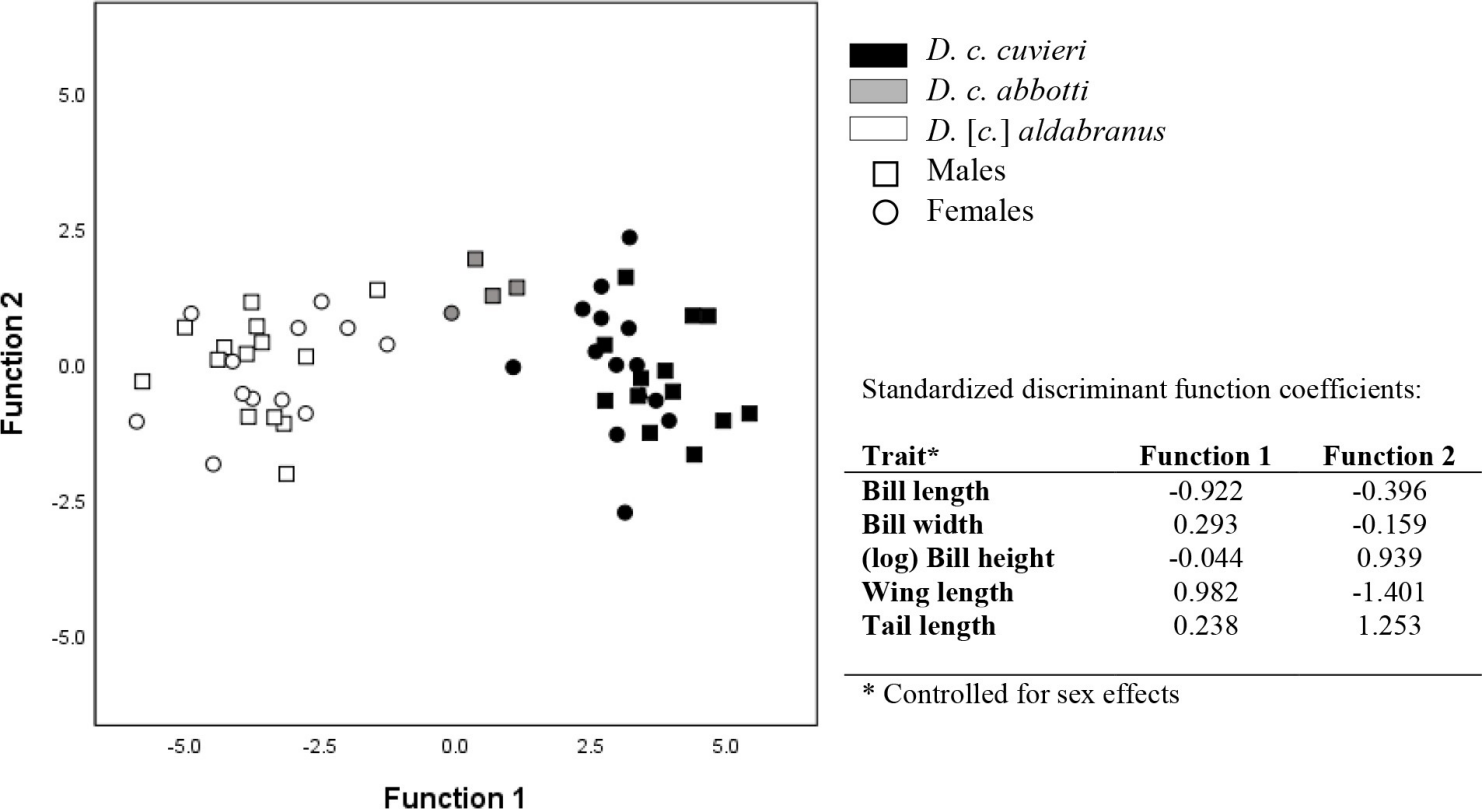
Plot of the two canonical functions resulting from the discriminant function analysis, with their coefficients for each of the morphological variables. Prior to the analysis, the data were corrected for sex. Different symbols indicate the different sexes.

Discriminant function analysis revealed the presence of morphological differences between *D*. *c*. *cuvieri*, *abbotti* and *aldabranus* (Wilks’ lambda = 0.066, Chi-squared = 141.11, df = 10, p<0.001; Fig 4). Two discriminant functions were found accounting for 100% of variation, with the first function accounting for 98.7% of variation between groups. Overall, the proportions of individuals correctly classified into their original groups were *D*. *c*. *cuvieri* = 96.3%, *D*. *c*. *abbotti* = 100% and *D*. [*c*.] *aldabranus* = 92.3%. The wings and tail of *Dryolimnas* [*c*.] *aldabranus* are the shortest, followed by *D*. *c*. *abbotti* and *D*. *c*. *cuvieri*, respectively. *Dryolimnas* [*c*.] *aldabranus* has a significantly longer bill than the other two subspecies (Table 3).

**Table 3:** Subspecies differences (between *D*. *c*. *cuvieri*, *abbotti* and *aldabranus*) for different morphological measurements.

		Mean ± SD			Subspecies			Covariate: Sex			Sex * subspecies		
Parameter	Sex	*D*.[*c*.]*aldabranus*	*D*.*c*.*abbotti*	*D*.*c*.*cuvieri*	*F*	*d*.*f*.	*p*	*F*	*d*.*f*.	*p*	*F*	*d*.*f*.	*p*
**(a) Wing length (mm)**	Male	116.66 ± 7.03 (n = 15)	135.25 ± 2.06 (n = 4)	154.43 ± 7.39 (n = 14)	**177.58**	**2**	**<0.001**	0.51	1	0.48	**3.14**	**2**	**0.051**
	Female	118.58 ± 4.75 (n = 13)	135.67 ± 2.89 (n = 3)	147.67 ± 7.98 (n = 15)									
**(b) Tail length (mm)**	Male	32.71 ± 5.77 (n = 15)	54.16 ± 3.41 (n = 5)	61.93 ± 5.33 (n = 14)	**175.49**	**2**	**<0.001**	0.71	1	0.4	2.42	2	0.097
	Female	36.96 ± 5.32 (n = 13)	57.8 ± 3.47 (n = 3)	59.67 ± 7.26 (n = 15)									
**(c) Bill length (mm)**	Male	45.79 ± 2.57 (n = 15)	42.48 ± 3.16 (n = 5)	42.0 ± 2.83 (n = 14)	**15.77**	**2**	**<0.001**	**10.26**	**1**	**<0.001**	1.5	2	0.23
	Female	43.86 ± 2.83 (n = 13)	37.0 ± 4.51 (n = 3)	40.34 ± 2.32 (n = 15)									
(d) Bill width (mm)	Male	5.94 ± 0.47 (n = 15)	6.06 ± 0.49 (n = 5)	5.94 ± 0.58 (n = 15)	1.66	2	0.2	**8.88**	**1**	**0.004**	2.38	2	0.1
	Female	5.28 ± 0.73 (n = 13)	5.27 ± 0.15 (n = 3)	5.85 ± 0.44 (n = 14)									
(e) Bill height (log) (mm)	Male	8.93 ± 0.90 (n = 14)	9.53 ± 0.54 (n = 4)	9.47 ± 0.70 (n = 15)	2.34	2	0.1	3.12	1	0.08	0.16	2	0.86
	Female	8.52 ± 0.91 (n = 12)	8.8 ± — (n = 1)	9.2 ± 0.74 (n = 15)									

(A) wing length, (B) tail length, (C) bill length, (D) bill width and (E) (log)bill height, with sex analysed as covariate (along with the interaction between subspecies and sex). The values shown are results from final models where the subspecies*sex and sex were eliminated respectively, if non-significant (statistically significant parameters are shown in bold).

## Discussion

*Dryolimnas* is a rare example of ability and inability to fly within what is currently considered a single species [23]. Our results suggest that the flightless *Dryolimnas* [*c*.] *aldabranus* has undergone an extended period of evolution on Aldabra (accumulating up to 2.1% absolute divergence from the Madagascar population, over an estimated 80,000–130,000 years). Loss of flight must have evolved rapidly, in less than 130,000 years based on our estimations, which concords with inferences made from subfossils [28]. This places the Aldabra rail well within the league of most rapid documented flight loss cases [9,13,15,41]. However, the enigma presented by its flightlessness does not seem fully explained by the speed of flight loss alone: there appear to exist younger fully flightless bird lineages worldwide, whether we consider date estimates alone (the flightless *Porzana palmeri* is estimated to have diverged within the past 125,000 years from its volant sister species, *Porzana pusilla*; [15]), or take genetic divergence as a proxy for time (flightless *Rallus sylvestris* showing only 1.2% absolute divergence in Cytb from volant *Rallus philippensis* [41]). Rather, the existence of a flightless (and poorly volant) subspecies within an otherwise volant species is primarily accounted for by the taxonomic status assigned to these taxa. To our knowledge, all other flightless bird lineages whose closest relatives are volant currently have full species status, even though the degree of genetic divergence encountered is sometimes lower (e.g. the *Rallus sylvestris-philippensis* case above) than the highest absolute divergences encountered here, of 2.1% between flightless *D*. [*c*.] *aldabranus* and volant *D*. *c*. *cuvieri*, and 1.7% between *D*. [*c*.] *aldabranus* and poorly volant *D*. *c*. *abbotti*.

The rapid evolutionary change associated with such cases of flight loss, despite low genetic divergence, is generally believed to be driven by selection rather than genetic drift, as maintaining such traits as energetically costly flight muscles [11,58] is presumably unnecessary in an environment in which the ability to fly confers little or no selective advantage [5,15,41]. Indeed the energetic savings (and fat storage) associated with reduced flight musculature could be an adaptation to survive periods of food and water scarcity in Aldabra's long dry season [11]. Hume *et al*. [28,59] propose that *D*. [*c*.] *aldabranus* was already flightless by 100,000 ybp, as a fossil *D*. [*c*.] *aldabranus* tarsometatarsus from this period (found on Point Hodoul, Grande Terre) measures within the size range of the present flightless population of *D*. [*c*.] *aldabranus*. Flightlessness may result from variations in development of several physical traits [60], such as underdeveloped pectoral muscles, asymmetry of wings (both confirmed to be the case for *D*. [*c*.] *aldabranus* [see 12]), increases in body mass, and changed proportions in skeletal elements [60,61]. Changes in skeletal elements and body mass, associated with the evolution towards flightlessness, may also be present in the subspecies of *D*. *cuvieri*, but this remains to be tested. Mass differences were not possible to examine using museum skin specimens. Flightlessness can also be associated with shortened flight feathers (i.e, reduced wing and tail length [8,62]. Our finding that *D*. [*c*.] *aldabranus* has shorter wings and tail than *D*. *c*. *abbotti* and *D*. *c*. *cuvieri* supports reports from Ridgway and Abbott [26]) and Benson [22], but not Wanless [11]. Bill size may also evolve due to changes in foraging ecology [e.g., 63] and the longer bill of *D*. [*c*.] *aldabranus* (see below), also found by Benson [22], might be an adaptation to foraging for crabs/prey in limestone crevices. Concomitant evolution of flightlessness potentially facilitated this adaptation, as weight restrictions became less critical with the loss of flight. Male *D*. *c*. *cuvieri* generally had a longer bill than females (independent-samples t-test; p = 0.009), and a longer bill length of *D*. [*c*.] *aldabranus* than *D*. *c*. *abbotti* and *D*. *c*. *cuvieri* was found in both sexes (all p<0.006, except for male *D*. [*c*.] *abbotti* which showed a borderline difference of p = 0.07 with *D*. *c*. *aldabranus*).

Morphological changes are frequently due to selection on a limited number of loci. In the flightless Galapagos cormorant (*Phalacrocorax harrisi*), a series of candidate function-altering genetic variants was found that likely contributed to the evolution of flightlessness [60]. Given the gradations of rapid evolution towards flightlessness (and genetic differentiation) documented here in *Dryolimnas*, and the fact that both *D*. [*c*.] *aldabranus* and *D*. *c*. *cuvieri* are still extant, a genome-wide study should provide further insights into the adaptive evolution of flightlessness.

### Colonisation patterns of *D*. [*c*.] *aldabranus*

Ancestors of *D*. [*c*.] *aldabranus* could have reached Aldabra via multiple colonisation events, which would explain the number of haplotypes within the living and historical populations of the Aldabra group relative to Madagascar, but is biogeographically puzzling. Viewing the two main genetic groupings (Native South Aldabra and Native North Aldabra; Figs 2 and 3) as independent colonisations, it is curious that they have managed to remain separate lineages throughout the period since arrival. Aldabra has undergone numerous rapid and major changes in geography in the last 200,000 years, prior to the atoll’s configuration today [59,64]. It may or may not have consisted of multiple islands at the time rails first colonised, and may have been a single island at least once since then. Regardless of precise history of changes in island geography and rail distribution, any scenario of two or more colonisations causing the genetic diversity of the Native South and North Aldabra populations we uncovered, needs to incorporate the inability of colonising populations to establish or introgress throughout the island or atoll, which is difficult to fully explain, assuming that at least one colonisation was of Madagascan origin, and fully volant upon arrival.

The alternative scenario of a single colonisation of Aldabra remains plausible given the lack of support for nodes generating the non-monophyly of the Aldabra populations (Fig 2), and the inability of the SH test to reject monophyly. For a single colonisation of the Aldabra group to explain the observed number and divergence of haplotypes, haplotype divergence of the small colonist population must have been as high, or higher, than it is across Madagascar today (Fig 3), at least for the CR (S4 Appendix). This, however, is not inconsistent with avian population histories in Madagascar. Humans arrived in Madagascar only 1500–2300 years ago [65], and have had a profound impact on native habitats [e.g., 66–68]. Recent (pre-human) avian extinctions and loss of genetic diversity in Madagascar have been speculated for various bird groups [see 69 for a review].

Whether one or multiple colonisations gave rise to *D*. *cuvieri* of the Aldabra group, the fact that rails native to South Aldabra are more closely related to those of Assumption than of North Aldabra (Fig 3, S4 Appendix) supports inter-island colonisation between Assumption and Aldabra. However, whether propagules from Madagascar colonised Aldabra via Assumption, or vice versa, is unclear.

Genetic differences of *D*. [*c*.] *aldabranus* between islands of Aldabra atoll itself are substantial, despite the lack of significant support for nodes in our data. It has been proposed that the restricted dispersal ability of *D*. [*c*.] *aldabranus* could limit gene flow between islands, resulting in inter-island genetic differences [30]. The probable genetic distinction of Île aux Cèdres rails from those on Malabar, Malabar* and Polymnie matches their distinctive morphological measurements [23,25] and plumage [70], but differences were not observed by [25]. Furthermore, a high differentiation in microsatellites was found in rails on Île aux Cèdres and Polymnie, with respect to each other and to Malabar rails [30].

The separation of what are now the Native South and North Aldabra populations likely began when Aldabra presented a very different geographic setting from the one we know today, the present island configuration possibly being as recent as 5000–7000 years [59,64,71]. The isolation of the northern and southern islands of the atoll probably explains how the Native North and Native South lineages have remained isolated since then. Île aux Cèdres is a small (0.5 km^2^) lagoon islet, closest to Grande Terre (distance: 253m) and separated from Malabar by a *ca*. 15m wide, deep channel (Fig 1). It is unlikely that flightless rails (at present sea level) would cross this channel. Île aux Cèdres’ proximity to Grande Terre, where rails were presumably extirpated before the late 1800s, raises the possibility that these rails were a remnant of the extinct Grande Terre population. The fact that Île aux Cèdres rails cluster more closely to the original Picard rails than to those of other Aldabra islands appears counterintuitive as Picard lies on the other side of the atoll (Fig 1). However, the extinct Grande Terre rails may have resembled the extinct Picard rails, as the channels separating Picard and Grande Terre, are shallow (maximum 5m depth; [72]) and contain several islets, making gene flow between rails on these islands probable. In contrast, the channels between Grande Terre and Malabar (Passe Hoareau, *ca*. 15m depth), and between Picard and Polymnie (Main Channel, ca. 20m depth; [72]) are considerably deeper, with fewer ‘stepping stones’. Such barriers are expected to have maintained these populations isolated in recent times (<7000 ybp, and conceivably in earlier sea-level lowstands), with significantly reduced gene flow.

Our study provides a good example of the value of museum collections in understanding biogeographic and evolutionary history, and in informing conservation management of closely related extant species. Genetic and morphological data from museum specimens of extinct rail populations were essential to outline the evolutionary pathway of populations and identify appropriate conservation recommendations for *D*. [*c*.] *aldabranus*. Our understanding of extant genetic diversity would have been greatly impoverished without access to extinct genetic diversity archived in museum specimens.

### Conservation management of *D*. [*c*.] *aldabranus*

Phylogenetic data, combined with data on morphology and behaviour, is a useful basis upon which to assess whether a population is sufficiently evolutionarily distinct from others to be treated as a separate conservation management unit. Despite morphological similarities between *Dryolimnas* on Aldabra and Madagascar, species boundaries have long been debated as it is argued that the populations must have been isolated for considerable time for flightlessness of the Aldabra population to have evolved. The surprisingly high genetic divergence and marked morphological differences of the Aldabra and Assumption subspecies from those of Madagascar, warrant the management, protection and assessment of the remaining Aldabra population as distinct from the Madagascar population. The small population size of *D*. [*c*.] *aldabranus* and its history of local extirpation, combined with the fact that it has evolved flightlessness and is consequently more vulnerable, increases the need for appropriate conservation management.

*Dryolimnas cuvieri* is currently Red-Listed as ‘Least Concern’ [73]. Unlike the common *D*. *c*. *cuvieri* on Madagascar, however, the restricted range, small population size and an ongoing threat from introduced cats on Grande Terre make *D*. [*c*.] *aldabranus* much more vulnerable to extinction. A Red List status that actually applies to a widely distributed, volant and less threatened subspecies is inappropriate and could compromise conservation management [74]. We therefore recommend re-assessment of *Dryolimnas cuvieri* subspecies by the IUCN to better reflect threat status. Given our results, *D*. [*c*.] *aldabranus* should at least be treated as a subspecies Vulnerable to extinction, based on IUCN criteria B and D2 (S5 Appendix).

Some authorities have already treated *Dryolimnas* [*c*.] *aldabranus* as a full species, distinct from *D*. *c*. *cuvieri* [e.g., 36]. The genetic divergence we uncover here certainly supports this view; to our knowledge, it is greater than that observed in all other such cases of closely-related volant-versus-flightless rail taxa, all of which are currently treated as full species. However, multiple species definitions are possible, with no single one being universally accepted [75,76]. Due to lack of significant support for nodes within *D*. *cuvieri*, our genetic data alone do not allow us to advocate treating *D*. [*c*.] *aldabranus* as a full species from a cladistic perspective. However, obtaining affordable and consistent sequence data from numerous historical samples necessarily restricted the length of sequence data obtained. It is conceivable that *D*. [*c*.] *aldabranus* will prove monophyletic based on genome-wide data, since our SH test showed that a hypothesis of monophyly cannot be rejected. Furthermore, regardless of whether or not *D*. [*c*.] *aldabranus* is monophyletic, it may well be a full species under a biological species concept. We remain open to such a decision being made by taxonomic authorities should they consider there to be sufficient justification.

In view of applying our results to conservation management and given the situation on the ground, we recommend the following conservation management measures:

1. Efforts to reinforce *D*. [*c*.] *aldabranus*’ population should consider substantial genetic divergence between Native North and South Aldabra. Unfortunately, it is probable that the last remnant of the Native South Aldabra population (Île aux Cèdres) is now extinct. Nonetheless, it is possible that a few individuals are still present and, until this possibility is ruled out, translocation of individuals of Native North Aldabra origin to Île aux Cèdres (or Grande Terre) should be avoided. Performed prematurely, such a translocation risks extinguishing Native South Aldabra rail genetic diversity through hybridisation;
2. It is likely that the introduction of cats caused the extirpation of the original *D*. [*c*.] *aldabranus* subpopulations on Picard, Grande Terre and possibly also on Île aux Cèdres. Cats could easily colonise Aldabra's other islands from Grande Terre, so it is important to eradicate cats as soon as is logistically feasible on this large and remote island.
3. Rats may also compromise breeding success of *D*. [*c*.] *aldabranus*, although the effects may be limited (but not absent) as this species has been reported to be able to defend itself against, and even kill, rats [see 77]. Nevertheless, for broad conservation reasons, planning for a rat eradication programme is underway and should be prioritized; however, during eradication it will be essential to maintain a captive population of rails from as broad a geographic range as possible across Polymnie and Malabar to safeguard the genetic variation they present.
4. Translocation of *D*. [*c*.] *aldabranus* should be considered to other islands in the Aldabra group (e.g., Assumption) and Western Indian Ocean preferably only when rat- and cat-free. Translocated groups should contain individuals from both Polymnie and Malabar.

As the last extant flightless bird in the Western Indian Ocean, the Aldabra white-throated rail has unique conservation significance. Our research sheds new light both on the phylogeny and evolution of flightlessness in *Dryolimnas*, and on its colonisation history, with important implications for conservation management. The flightless *D*. [*c*.] *aldabranus* is clearly on a separate evolutionary trajectory from the volant *D*. *c*. *cuvieri*. Its evolutionary uniqueness, based on genetic and morphological divergence, warrants treating *D*. [*c*.] *aldabranus* as an independent conservation management unit.

## Supporting information

S1 Appendix Detailed information for the historical specimens used in this study. (DOC)

S2 Appendix Molecular methods. (DOC)

S3 Appendix (1) Phylogenetic tree from Fig 2 (Yule speciation prior, 30 million generations), with the outgroups included. (2) Dated cladogram applying Coalescent-Inversegamma speciation prior, 30 million generations. (3) Dated cladogram applying Coalescent-Uniform speciation prior, 10 million generations.
(DOC)

S4 Appendix Median-joining haplotype networks for each of the markers used in this study. (DOCX)

S5 Appendix Evaluation of *D*. [*c*.] *aldabranus* classification against IUCN criteria. (DOCX)
