# Correction: Rapid loss of flight in the Aldabra white-throated rail

**DOI:** 10.1371/journal.pone.0242726

**Published:** 2020-11-16

**Authors:** Janske van de Crommenacker, Nancy Bunbury, Hazel A. Jackson, Lisa J. Nupen, Ross Wanless, Frauke Fleischer-Dogley, Jim J. Groombridge, Ben H. Warren

There is an error in affiliation 6 for author Ben H. Warren. The correct affiliation 6 is: Institut de Systématique, Evolution, Biodiversité (ISYEB), Muséum National d'Histoire Naturelle, Sorbonne Universités, CNRS, EPHE, UA, Paris, France.

In the ‘Colonisation patterns of *D*. *[c*.*] aldabranus’* subsection of the Discussion, there is an error in the third sentence of the fifth paragraph. The correct sentence is: Ile aux Cèdres is a small (0.5 km^2^) lagoon islet, closest to Grande Terre (distance: 253m) and separated from Malabar (via Grande Terre) by a *ca*. 15m wide, deep channel (Fig 1).

**Fig 1:**
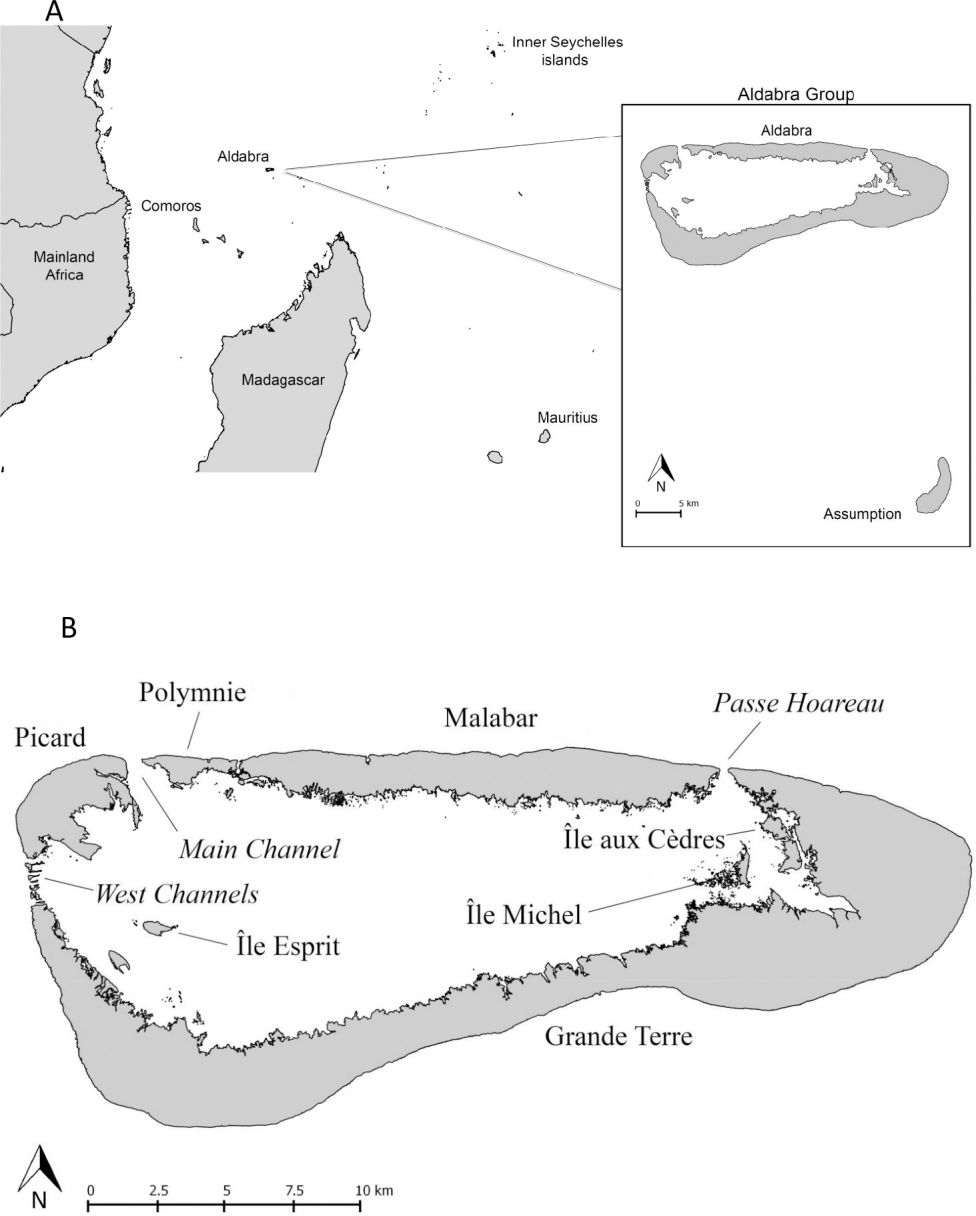
(A) Western Indian Ocean with Madagascar, Aldabra Atoll and Assumption Island (the latter two enlarged in the inset), and (B) the islands of Aldabra Atoll, of which Picard, Malabar and Polymnie are populated by *D*. *[c*.*] aldabranus*, as was Île aux Cèdres until recently.

The upper and lower panels of Fig 1 are missing labels. Please see the correct Fig 1 here.
