# Supplementary material for: Rapid loss of flight in the Aldabra white-throated rail
Source: PLoS One. 2019 Dec 23;14(12):e0226064. doi: 10.1371/journal.pone.0226064 (PMC6927662; doi:10.1371/journal.pone.0226064)
Supplement: S1 Appendix — (DOC) [file pone.0226064.s001.doc]

**Supporting information**

**S1 Appendix.** **Detailed information for the historical specimens used in this study.**

NHM = Natural History Museum in Tring, UK; AMNH = American Museum of Natural History in New York, USA); USNM = Smithsonian Institution, National Museum of Natural History, Washington DC, USA).

| **Subspp.** | **Genetics ID (Fig. 2)** | **Museum Label** | **Collection date** | **Museum** | **Sample location** | **Lab ID (if included in genetic analyses)** | **Morpho-logical analyses?** |
| --- | --- | --- | --- | --- | --- | --- | --- |
| **Samples included in this studies' genetic AND morphological analyses** | | | | | | | |
| *D.* [*c.*] *aldabranus* | *D. [c.] aldabranus* M32 | 1968.43.102 | 12/03/1968 | NHM | Aldabra Atoll, island unknown | AldaRail32 | X |
| *D.* [*c.*] *aldabranus* | *D. [c.] aldabranus* M33 | 1906.12.28.14 | 10/1906 | NHM | Aldabra Atoll, island unknown | AldaRail33 | X |
| *D.* [*c.*] *aldabranus* | *D. [c.] aldabranus* M34 | 1977.10.70 | 18/05/1972 | NHM | Aldabra Atoll, Malabar | AldaRail34 | X |
| *D.* [*c.*] *aldabranus* | *D. [c.] aldabranus* M35 | AMNH545395 | 14/10/1903 | ANMH | Aldabra Atoll, Picard (pre-extinction) | AldaRail35 | X |
| *D.* [*c.*] *aldabranus* | *D. [c.] aldabranus* M36 | AMNH545396 | 14/10/1903 | ANMH | Aldabra Atoll, Picard (pre-extinction) | AldaRail36 | X |
| *D.* [*c.*] *aldabranus* | *D. [c.] aldabranus* M37 | AMNH545397 | 14/10/1903 | ANMH | Aldabra Atoll, Picard (pre-extinction) | AldaRail37 | X |
| *D. c. abbotti* | *D. c. abbotti* 1 | 1906.12.21.139 | 12/03/1906 | NHM | Assumption | AssRail1 | X |
| *D. c. abbotti* | *D. c. abbotti* 2 | 1906.12.21.141 | 12/03/1906 | NHM | Assumption | AssRail2 | X |
| *D. c. abbotti* | *D. c. abbotti* 3 | 1906.12.21.142 | 12/03/1906 | NHM | Assumption | AssRail3 | X |
| *D. c. abbotti* | *D. c. abbotti* 5 | 1906.12.21.140 | 12/03/1906 | NHM | Assumption | AssRail5 | X |
| *D. c. cuvieri* | *D. c. cuvieri* 100 | 1931.8.18.1765 | 27/11/1930 | NHM | N Madagascar, Bezona, East of Ambanja | MadRail100 | X |
| *D. c. cuvieri* | *D. c. cuvieri* 101 | 1931.8.18.1004 | 02/11/1930 | NHM | N Madagascar, Andranofanjava | MadRail101 | X |
| *D. c. cuvieri* | *D. c. cuvieri* 102 | 1931.8.18.1759 | 24/11/1929 | NHM | SW Madagascar, Befandriana | MadRail102 | X |
| *D. c. cuvieri* | *D. c. cuvieri* 103 | 1931.8.1.8.1757 | 22/08/1930 | NHM | N. Madagascar 1 Day West of Andapa | MadRail103 | X |
| *D. c. cuvieri* | *D. c. cuvieri* 105 | 1969.48.101 | n/a | NHM | Centr. Madagascar, District de Rogez, foret orientale | MadRail105 | X |
| *D. c. cuvieri* | *D. c. cuvieri* 106 | 1889.11.3.72 | 3/3/1881 | NHM | N. Madagascar, Tsikoza, Ankafana | MadRail106 | X |
| *D. c. cuvieri* | *D. c. cuvieri* 107 | 1931.8.18.1002 | 13/08/1929 | NHM | SE Madagascar, Ivohibe | MadRail107 | X |
| *D. c. cuvieri* | *D. c. cuvieri* 108 | 1931.8.18.1760 | 26/10/1930 | NHM | N. Madagascar, Mt. D'Ambre, | MadRail108 | X |
| *D. c. cuvieri* | *D. c. cuvieri* 109 | 1931.8.18.1764 | 03/01/1931 | NHM | N. Madagascar, Bezona, East of Ambanja | MadRail109 | X |
| **Samples NOT included in this studies' genetic analyses, but used for morphological analyses** | | | | | | | |
| *D.* [*c.*] *aldabranus* | -- | 1939.12.9.2939 | 08/07/1906 | NHM | Aldabra Atoll, specific location unknown (likely Picard) |  | X |
| *D.* [*c.*] *aldabranus* | -- | 1906.12.21.144 | 13/03/1906 | NHM | Aldabra Atoll, specific location unknown (likely Malabar) |  | X |
| *D.* [*c.*] *aldabranus* | -- | 78.12.31.5 | n/a | NHM | Aldabra Atoll, specific location unknown |  | X |
| *D.* [*c.*] *aldabranus* | -- | 1906.12.21.143 | 15/03/1906 | NHM | Aldabra Atoll, specific location unknown (likely Malabar) |  | X |
| *D.* [*c.*] *aldabranus* | -- | 1904.12.4.6 | Sept-Nov 1906 | NHM | Aldabra Atoll, specific location unknown |  | X |
| *D.* [*c.*] *aldabranus* | -- | 1907.12.4.5 | Sept-Nov 1906 | NHM | Aldabra Atoll, specific location unknown |  | X |
| *D.* [*c.*] *aldabranus* | -- | 1939.12.9.2938 | 08/07/1906 | NHM | Aldabra Atoll, specific location unknown (likely Picard) |  | X |
| *D.* [*c.*] *aldabranus* | -- | AMNH545384 | 08/07/1906 | ANMH | Aldabra Atoll, specific location unknown |  | X |
| *D.* [*c.*] *aldabranus* | -- | AMNH545385 | 08/07/1906 | ANMH | Aldabra Atoll, specific location unknown |  | X |
| *D.* [*c.*] *aldabranus* | -- | AMNH545386 | 08/07/1906 | ANMH | Aldabra Atoll, specific location unknown |  | X |
| *D.* [*c.*] *aldabranus* | -- | AMNH545387 | 08/07/1906 | ANMH | Aldabra Atoll, specific location unknown |  | X |
| *D.* [*c.*] *aldabranus* | -- | AMNH545388 | n/a | ANMH | Aldabra Atoll, specific location unknown |  | X |
| *D.* [*c.*] *aldabranus* | -- | AMNH545389 | n/a | ANMH | Aldabra Atoll, specific location unknown |  | X |
| *D.* [*c.*] *aldabranus* | -- | AMNH545390 | n/a | ANMH | Aldabra Atoll, specific location unknown |  | X |
| *D.* [*c.*] *aldabranus* | -- | AMNH545391 | n/a | ANMH | Aldabra Atoll, specific location unknown |  | X |
| *D.* [*c.*] *aldabranus* | -- | AMNH545392 | n/a | ANMH | Aldabra Atoll, specific location unknown |  | X |
| *D.* [*c.*] *aldabranus* | -- | AMNH545393 | 01/10/1903 | ANMH | Aldabra Atoll, specific location unknown |  | X |
| *D.* [*c.*] *aldabranus* | -- | AMNH545394 | 01/10/1903 | ANMH | Aldabra Atoll, specific location unknown |  | X |
| *D.* [*c.*] *aldabranus* | -- | AMNH545398 | 28/09/1903 | ANMH | Aldabra Atoll, Picard |  | X |
| *D.* [*c.*] *aldabranus* | -- | AMNH545399 | 02/09/1903 | ANMH | Aldabra Atoll, Picard |  | X |
| *D.* [*c.*] *aldabranus* | -- | AMNH545400 | 02/10/1903 | ANMH | Aldabra Atoll, Picard |  | X |
| *D.* [*c.*] *aldabranus* | -- | AMNH545401 | 14/10/1903 | ANMH | Aldabra Atoll, Picard |  | X |
| *D.* [*c.*] *aldabranus* | -- | AMNH545402 | 14/10/1903 | ANMH | Aldabra Atoll, Picard |  | X |
| *D.* [*c.*] *aldabranus* | -- | USNM128833 | 04/10/1892 | USNM | Aldabra Atoll, specific location unknown |  | X |
| *D.* [*c.*] *aldabranus* | -- | USNM128830 | 01/10/1892 | USNM | Aldabra Atoll, specific location unknown |  | X |
| *D.* [*c.*] *aldabranus* | -- | USNM128834 | 10/10/1892 | USNM | Aldabra Atoll, specific location unknown |  | X |
| *D.* [*c.*] *aldabranus* | -- | USNM128837 | 18/10/1892 | USNM | Aldabra Atoll, specific location unknown |  | X |
| *D.* [*c.*] *aldabranus* | -- | USNM128836 | 18/09/1892 | USNM | Aldabra Atoll, specific location unknown / Assumption? |  | X |
| *D. c. abbotti* | *D. c. abbotti* 4  (poor quality sequence) | 1906.12.21.138 | 12/03/1906 | NHM | Assumption | AssRail4 | X |
| *D. c. abbotti* | -- | USNM128827 | 18/09/1892 | USNM | Assumption |  | X |
| *D. c. abbotti* | -- | USNM128828 | 18/09/1892 | USNM | Assumption |  | X |
| *D. c. abbotti* | -- | USNM128829 | 18/09/1892 | USNM | Assumption |  | X |
| *D. c. cuvieri* | *D. c. cuvieri* 104  (poor quality sequence) | 1931.8.18.1761 | 24/11/1929 | NHM | SW Madagascar, Befandriana | MadRail104 | X |
| *D. c. cuvieri* | -- | Unreg. | 1888 | NHM | Madagascar, specific location unknown |  | X |
| *D. c. cuvieri* | -- | 1879.6.7.6 | n/a | NHM | Centr. Madagascar, Betsiles |  | X |
| *D. c. cuvieri* | -- | Unreg. | 1888 | NHM | Madagascar, specific location unknown |  | X |
| *D. c. cuvieri* | -- | 1931.8.18.1000 | 07/06/1930 | NHM | NE Madagascar, SW of Maroantsetra |  | X |
| *D. c. cuvieri* | -- | 1889.11.3.71 | 03/1881 | NHM | N. Madagascar, Ankafana |  | X |
| *D. c. cuvieri* | -- | 1889.11.3.73 | n/a | NHM | Madagascar, specific location unknown |  | X |
| *D. c. cuvieri* | -- | 1866.5.5.30 | n/a | NHM | E. Madagascar, Mohambo |  | X |
| *D. c. cuvieri* | -- | 1866.5.5.25 | n/a | NHM | E. Madagascar, Mohambo |  | X |
| *D. c. cuvieri* | -- | 1931.8.18.999 | 10/06/1930 | NHM | NE Madagascar, SW of Maroantsetra |  | X |
| *D. c. cuvieri* | -- | 1891.8.1.82 | n/a | NHM | Madagascar, specific location unknown |  | X |
| *D. c. cuvieri* | -- | 1882.2.27.112 | 14/03/1881 | NHM | N. Madagascar, Ankafana |  | X |
| *D. c. cuvieri* | -- | 1931.8.18.1003 | 09/08/1929 | NHM | SE Madagascar, Ivohibe |  | X |
| *D. c. cuvieri* | -- | 1891.8.1.80 | 20/10/1874 | NHM | Madagascar, Mare du Vinang Sambyre(?) |  | X |
| *D. c. cuvieri* | -- | 1891.8.1.83 | n/a | NHM | SE Coast Madagascar |  | X |
| *D. c. cuvieri* | -- | 1931.8.18.1767 | 26/03/1931 | NHM | Centr. Madagascar, Ambararatabé |  | X |
| *D. c. cuvieri* | -- | 1843.7.22.69 | n/a | NHM | Madagascar, specific location unknown |  | X |
| *D. c. cuvieri* | -- | 1931.8.18.1762 | 31/08/1930 | NHM | N. Madagascar, West of Andapa |  | X |
| *D. c. cuvieri* | -- | 1890.10.16.330 | n/a | NHM | Madagascar, specific location unknown |  | X |
| *D. c. cuvieri* | -- | 1969.43.39 | n/a | NHM | Madagascar, specific location unknown |  | X |
| *D. c. cuvieri* | -- | 1969.52.1065 | 23/11/1886 | NHM | NE Madagascar, Riviere Ivolina, Amajoustre |  | X |
| *D. c. cuvieri* | -- | 1931.8.18.1763 | 01/05/1929 | NHM | Centr. Madagascar, Foret Sianaka |  | X |
| *D. c. cuvieri* | -- | 1931.8.18.1758 | 13/08/1929 | NHM | SE Madagascar, Ivohibe |  | X |
| *D. c. cuvieri* | -- | 1931.8.18.1001 | 17/08/1929 | NHM | SE Madagascar, Ivohibe |  | X |
| *D. c. cuvieri* | -- | 1931.8.18.1766 | 23/01/1931 | NHM | N. Madagascar, East of Maromandia |  | X |
