# Supplementary material for: Rapid loss of flight in the Aldabra white-throated rail
Source: PLoS One. 2019 Dec 23;14(12):e0226064. doi: 10.1371/journal.pone.0226064 (PMC6927662; doi:10.1371/journal.pone.0226064)
Supplement: S2 Appendix — (DOC) [file pone.0226064.s002.doc]

S2 Appendix.

Molecular methods

*DNA extraction, PCR and Sequencing*

DNA from all blood samples and museum toepad specimens were extracted using the Isolate Genomic DNA Mini Kit (Bioline, UK). Samples were suspended in 400 μl Lysis buffer plus 40 μl (blood) or 25 μl (chopped museum toepads) of proteinase K and incubated at 55^o^C overnight (or until the toepad material had completely digested). DNA was washed through a spin column and suspended in 200 μl (blood) or 50 μl (museum specimens) elution buffer. Typically, 25μl PCRs were prepared, comprising the following reagents: 1μl DNA extract, 2μl of each of the forward and reverse primers (at 10μM dilution), 12.5μl My Taq HS Red Mix (Bioline, UK) and 7.5μl UV sterilised DNA grade distilled water (dH_2_O). PCR amplification of target regions was performed under the following cycling conditions: initial denaturation (1 min at 95°C); *n* cycles (marker-specific; Table 1) of 15 sec at 95°C, 15 sec at marker-specific temperature; Table 1, 10 sec at 95°C, and a final 10 min extension at 72°C. PCR results were verified by agarose gel electrophoresis with SybrSAFE staining and visualised using a Bio-Rad Gel Doc^TM^EZ Imager (viewing software: Bio-Rad Image Lab 3.0).

PCR products were purified and sequenced by Macrogen (Europe and South Korea). Sequence reads were manually checked and edited using the programmes FinchTV 1.4 (Geospiza), BioEdit 7.2.0 (1) and CodonCode Aligner 4.2.4 (CodonCode Corporation, Dedham, MA). Consensus sequences were aligned using the programme ClustalX 2.1.12 (2).

**References**

1. Hall TA. BioEdit: a user-friendly biological sequence alignment editor and analysis program for Windows 95/98/NT. In: Nucleic Acids Symposium Series. 1999. p. 95–98.

2. Larkin MA, Blackshields G, Brown NP, Chenna R, McGettigan PA, McWilliam H, et al. Clustal W and Clustal X version 2.0. Bioinformatics. 2007;23:2947–2948.
