# Supplementary material for: Rapid loss of flight in the Aldabra white-throated rail
Source: PLoS One. 2019 Dec 23;14(12):e0226064. doi: 10.1371/journal.pone.0226064 (PMC6927662; doi:10.1371/journal.pone.0226064)
Supplement: S3 Appendix — (1) Phylogenetic tree from Fig 2 (Yule speciation prior, 30 million generations), with the outgroups included. (2) Dated cladogram applying Coalescent-Inversegamma speciation prior, 30 million generations. (3) Dated cladogram applying Coalescent-Uniform speciation prior, 10 million generations. (DOC) [file pone.0226064.s003.doc]

S3 Appendix.

(1) Phylogenetic tree from Fig 2 (Yule speciation prior, 30 million generations), with the outgroups included. (2) Dated cladogram applying Coalescent-Inversegamma speciation prior, 30 million generations. (3) Dated cladogram applying Coalescent-Uniform speciation prior, 10 million generations.


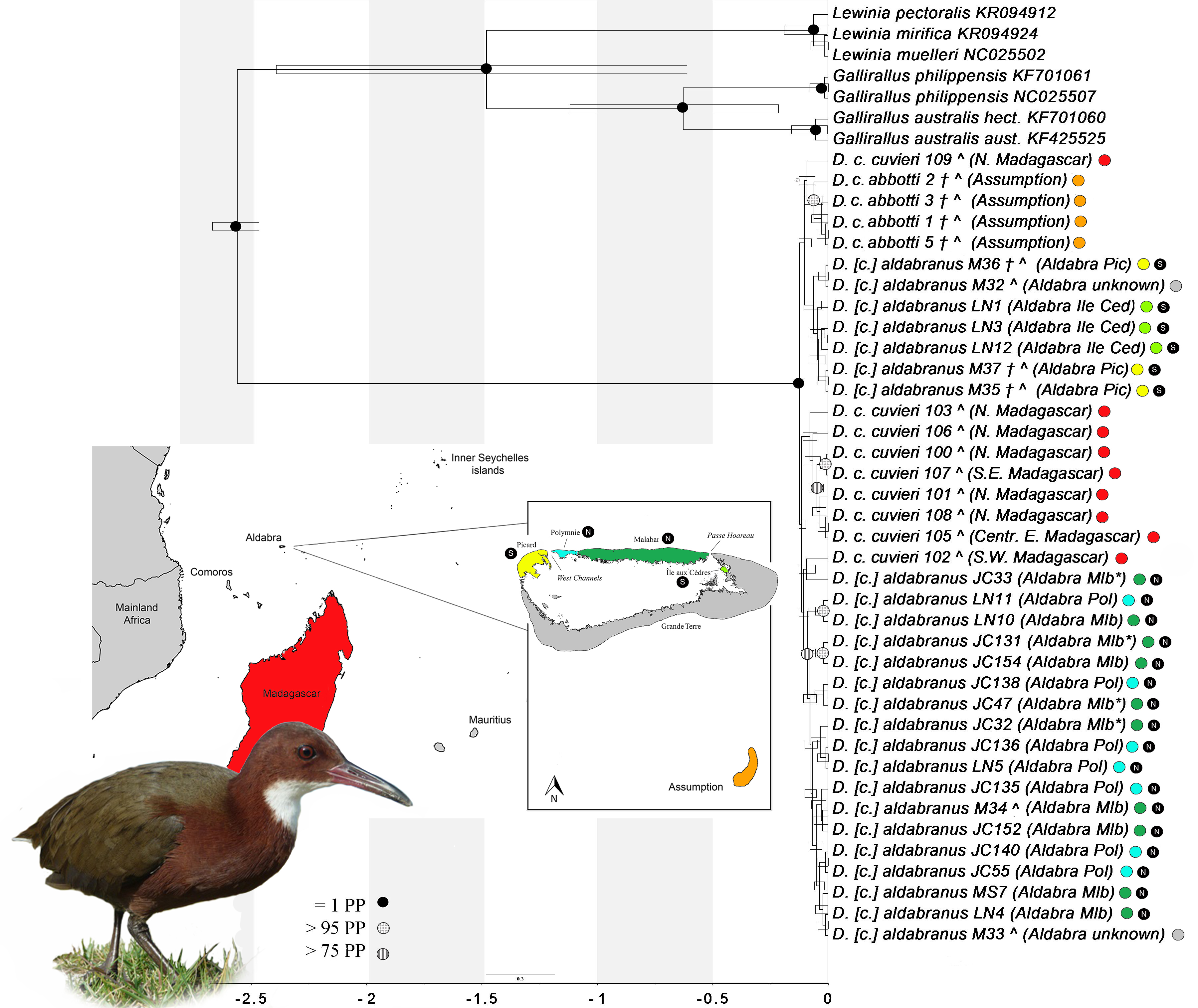
*(1) Phylogenetic tree Figure 2*


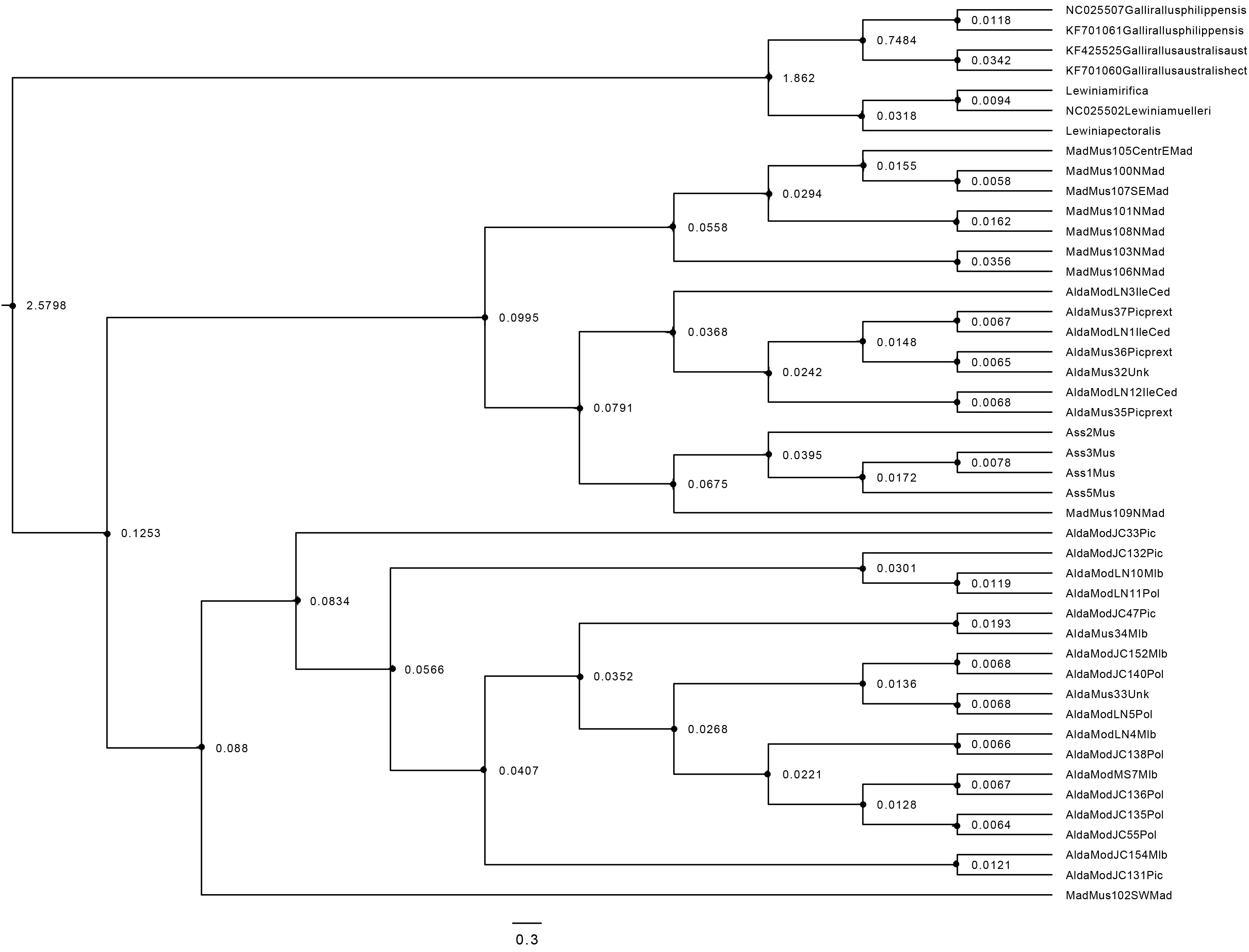
*(2) Dated cladogram applying Coalescent-Inversegamma speciation prior*

*(3) Dated cladogram applying Coalescent-Uniform speciation prior*

*
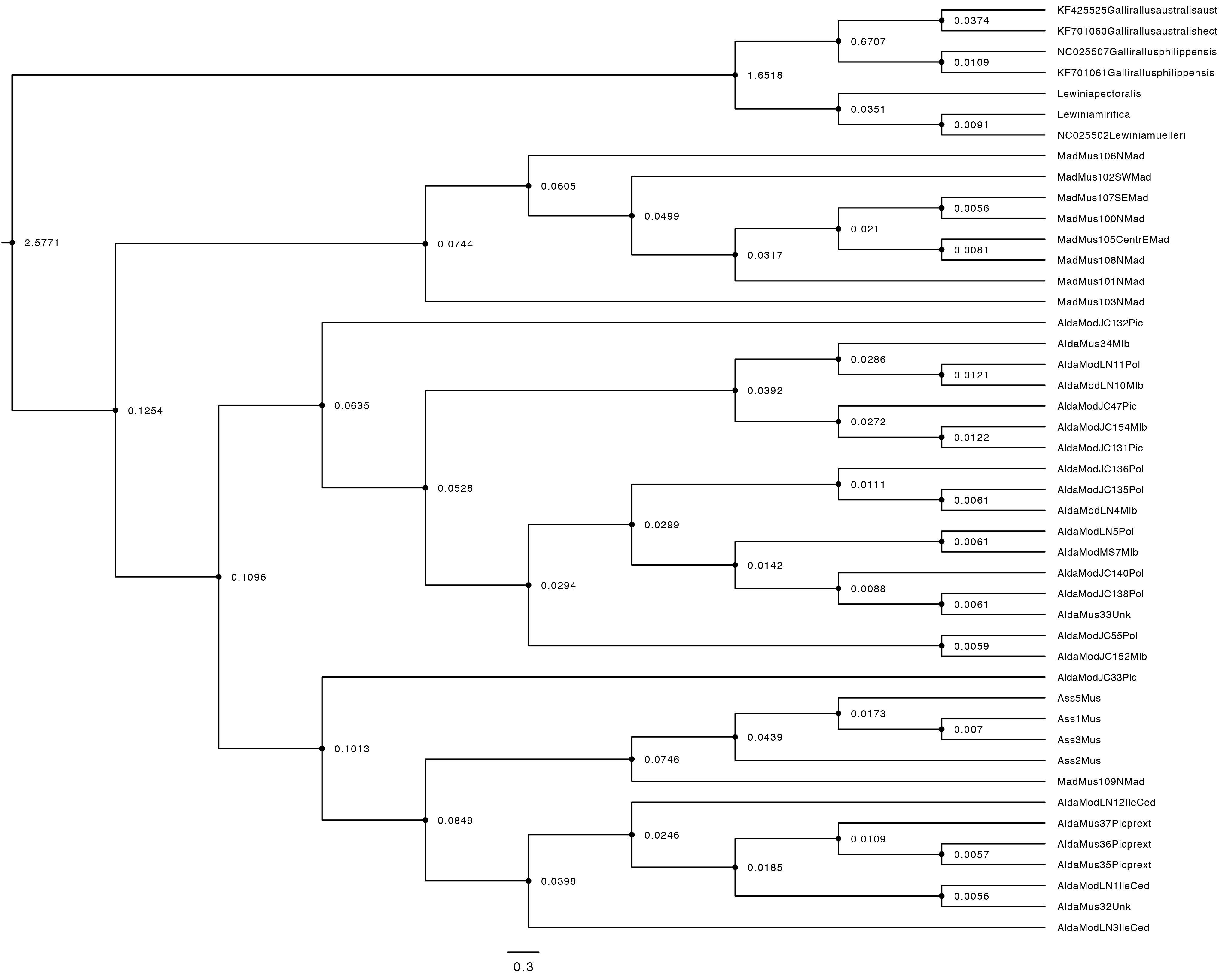
*
