# Supplementary material for: Rapid loss of flight in the Aldabra white-throated rail
Source: PLoS One. 2019 Dec 23;14(12):e0226064. doi: 10.1371/journal.pone.0226064 (PMC6927662; doi:10.1371/journal.pone.0226064)
Supplement: S4 Appendix — (DOCX) [file pone.0226064.s004.docx]

**S4 Appendix.**

**Median-joining haplotype networks for each of the markers used in this study**.

(a) Control Region and (b) Cytb. For the Aldabra rail, the Picard pre-extinction individuals, those from Île aux Cèdres and those caught from unknown locations are shown separately, whereas the individuals from Malabar and Malabar* are pooled.


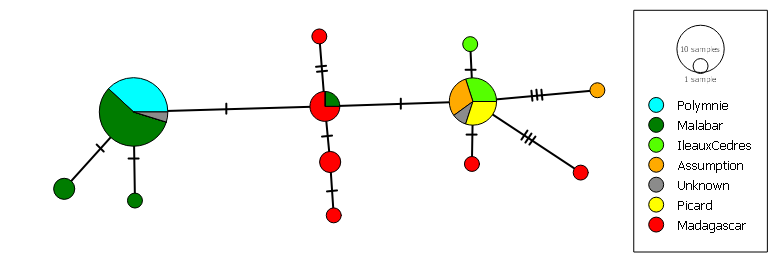

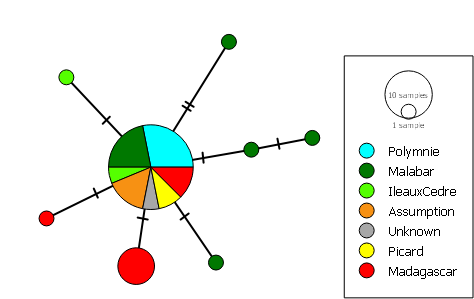


(a)

(b)
