# Supplementary material for: Rapid loss of flight in the Aldabra white-throated rail
Source: PLoS One. 2019 Dec 23;14(12):e0226064. doi: 10.1371/journal.pone.0226064 (PMC6927662; doi:10.1371/journal.pone.0226064)
Supplement: S5 Appendix — (DOCX) [file pone.0226064.s005.docx]

**S5 Appendix.**

**Evaluation of *D.* [*c.*] *aldabranus* classification against IUCN criteria**

*Dryolimnas* [*c.*] *aldabranus* is now classified by IUCN as being Least Concern. Classification within this category means that it has been evaluated against the IUCN criteria and does not qualify for Critically Endangered, Endangered, Vulnerable or Near Threatened. Widespread and abundant taxa are included in the category of Least Concern.

We propose *D.* [*c.*] *aldabranus* to be treated as Vulnerable. Classification in this category applies when the best available evidence indicates that it meets any of the following criteria (A to E), and it is therefore considered to be facing a high risk of extinction in the wild:

*A. Reduction in population size based on any of the following:*

*1. An observed, estimated, inferred or suspected population size reduction of ≥50% over the last 10 years or three generations, whichever is the longer, where the causes of the reduction are clearly reversible AND understood AND ceased, based on (and specifying) any of the following:*

*(a) direct observation*

*(b) an index of abundance appropriate to the taxon*

*(c) a decline in area of occupancy, extent of occurrence and/or quality of*

*habitat*

*(d) actual or potential levels of exploitation*

*(e) the effects of introduced taxa, hybridization, pathogens, pollutants,*

*competitors or parasites.*

**Not applicable**

*2. An observed, estimated, inferred or suspected population size reduction of*

*≥30% over the last 10 years or three generations, whichever is the longer, where*

*the reduction or its causes may not have ceased OR may not be understood OR*

*may not be reversible, based on (and specifying) any of (a) to (e) under A1.*

**Not applicable**

*3. A population size reduction of ≥30% projected or suspected to be met within the*

*next 10 years or three generations, whichever is the longer (up to a maximum of*

*100 years), based on (and specifying) any of (b) to (e) under A1.*

**Not applicable**

*4. An observed, estimated, inferred, projected or suspected population size*

*reduction of ≥30% over any 10 year or three generation period, whichever is*

*longer (up to a maximum of 100 years in the future), where the time period must*

*include both the past and the future, AND where the reduction or its causes may*

*not have ceased OR may not be understood OR may not be reversible, based*

*on (and specifying) any of (a) to (e) under A1.*

**Not applicable**

**In general, the population of *D.* [*c.*] *aldabranus* on Aldabra is currently considered to be stable. The reintroduced population on Picard has expanded to more than 2500 individuals since 1999 [1]. However, a subpopulation of *D.* [*c.*] *aldabranus* has most likely gone extinct recently on Île aux Cèdres, which was estimated to be at least 80 individuals in the mid-1970s [2,3]. *Dryolimnas* [*c.*] *aldabranus* was last confirmed to be present on Île aux Cèdres in 2000, when Wanless took blood samples of birds there [4]. This reduction, potentially due to the arrival of introduced predators (cats) or decline of habitat quality due to extended drought, warrants listing as Vulnerable under this criterion.**

*B. Geographic range in the form of either B1 (extent of occurrence) OR B2 (area of*

*occupancy) OR both:*

*1. Extent of occurrence estimated to be less than 20,000 km2, and estimates*

*indicating at least two of a-c:*

*a. Severely fragmented or known to exist at no more than 10 locations.*

*b. Continuing decline, observed, inferred or projected, in any of the following:*

*(i) extent of occurrence*

*(ii) area of occupancy*

*(iii) area, extent and/or quality of habitat*

*(iv) number of locations or subpopulations*

*(v) number of mature individuals.*

*c. Extreme fluctuations in any of the following:*

*(i) extent of occurrence*

*(ii) area of occupancy*

*(iii) number of locations or subpopulations*

*(iv) number of mature individuals.*

**Yes – 1a and 1b(iv) are applicable**

*2. Area of occupancy estimated to be less than 2,000 km2, and estimates indicating*

*at least two of a-c:*

*a. Severely fragmented or known to exist at no more than 10 locations.*

*b. Continuing decline, observed, inferred or projected, in any of the following:*

*(i) extent of occurrence*

*(ii) area of occupancy*

*(iii) area, extent and/or quality of habitat*

*(iv) number of locations or subpopulations*

*(v) number of mature individuals.*

*c. Extreme fluctuations in any of the following:*

*(i) extent of occurrence*

*(ii) area of occupancy*

*(iii) number of locations or subpopulations*

*(iv) number of mature individuals.*

**Yes – 2a and 2b(iv) are applicable. *Dryolimnas* [*c.*] *aldabranus* has an Extent of Occurrence of 37.2 km^2^ (i.e., the islands Picard (9.4 km^2^), Malabar (25.9 km^2^), Polymnie (1.9 km^2^) and a few satellite lagoon islets near Malabar) and meets the threshold for Endangered under criterion B1 (i.e., extent of occurrence estimated to be <100 km^2^), and its Area of Occupancy meets the threshold for Endangered (<500 km^2^) under criterion B2. Furthermore, the Île aux Cèdres subpopulation appears to have become recently extinct. The species’ range is currently considered stable, but there is a high possibility of continuing decline in the future as a result of the potential impacts of climate change (increasing drought frequency, sea level rise), and invasive predators such as cats and rats, in particular the threat of cats establishing on other islands with rails is very high. Additionally, it is likely found at less than five locations (see Criterion D). Therefore, it could potentially warrant listing as Endangered, or alternatively at least as Vulnerable under criteria B.**

*C. Population size estimated to number fewer than 10,000 mature individuals and*

*either:*

*1. An estimated continuing decline of at least 10% within 10 years or three*

*generations, whichever is longer, (up to a maximum of 100 years in the*

*future) OR*

**No, but see threats mentioned below**

*2. A continuing decline, observed, projected, or inferred, in numbers of mature*

*individuals AND at least one of the following (a-b):*

*a. Population structure in the form of one of the following:*

*(i) no subpopulation estimated to contain more than 1,000 mature*

*individuals, OR*

*(ii) all mature individuals in one subpopulation.*

*b. Extreme fluctuations in number of mature individuals.*

**No**

**The population size of this species has been estimated at ca. 2500 birds on Picard [1]. Previously published estimates for the other islands are outdated: intensive studies in the 1970s yielded population estimates of 7700 rails on Malabar, 270 on Polymnie and 80 on Île aux Cèdres [2]. New estimates are underway, but it is anticipated that the total population size is approximately or less than 10,000 mature individuals. At the moment there is no indication for a continuing decline, but threats such as the arrival / spread of introduced predators, decline of habitat quality due to extended drought frequency, or habitat loss due to sea level rise warrant listing as Vulnerable under this criterion.**

*D. Population very small or restricted in the form of either of the following:*

*1. Population size estimated to number fewer than 1,000 mature individuals.*

**No**

*2. Population with a very restricted area of occupancy (typically less than 20 km2)*

*or number of locations (typically five or fewer) such that it is prone to the effects*

*of human activities or stochastic events within a very short time period in an*

*uncertain future, and is thus capable of becoming Critically Endangered or even*

*Extinct in a very short time period.*

**Yes**

**The population size of *D.* [*c.*] *aldabranus* is larger than the criterion of 1,000 mature individuals. However, the number of locations where *D.* [*c.*] *aldabranus* is found is very small (three locations covering 37.2 km^2^), with subpopulations being confined to even smaller islands (i.e., the islands Picard (9.4 km^2^), Malabar (25.9 km^2^), Polymnie (1.9 km^2^). It could be questioned whether Aldabra Atoll itself is considered to be one location or if the four main constituent islands with subpopulations present (Malabar, Picard and Polymnie) are considered separate locations. Based on the potential threats listed under Criterion C in combination with this limited range, *D.* [*c.*] *aldabranus* may qualify as Vulnerable under criterion D2.**

*E. Quantitative analysis showing the probability of extinction in the wild is at least 10%*

*within 100 years.*

**Criterion E – No quantitative analysis of extinction risk has been conducted for this species. Therefore, it cannot be assessed against this criterion.**

Based on the above aspects, we propose *D.* [*c.*] *aldabranus* be up-listed to at least Vulnerable under criteria B and D2. the he ecology of the species, rather a discussion o

A

B

B B

A

B B

A

n = 34 8 34

C

**References**

1. Šúr M, van de Crommenacker J, Bunbury N. Assessing effectiveness of reintroduction of the flightless Aldabra rail on Picard Island, Aldabra Atoll, Seychelles. Conservation Evidence. 2013;10:80–4.

2. Huxley CR. The Aldabra rail. In Unpublished typescript, Seychelles Islands Foundation; 1982.

3. Wanless RM. The reintroduction of the Aldabra rail *Dryolimnas cuvieri aldabranus* to Picard Island, Aldabra Atoll. MSc thesis, University of Cape Town, South Africa; 2002.

4. Wanless RM, Cunningham J, Hockey PA, Wanless J, White RW, Wiseman R. The success of a soft-release reintroduction of the flightless Aldabra rail (*Dryolimnas* [*cuvieri*] *aldabranus*) on Aldabra Atoll, Seychelles. Biological Conservation. 2002;107:203–210.
